# Fabrication and Enhancement of the Gas Sensing Characteristics of Silicon Micropillar NH_3_ Sensors Based on MOF-808/rGO Nanocomposites at Room Temperature

**DOI:** 10.3390/s26103216

**Published:** 2026-05-19

**Authors:** Haoyue Wang, Shaolun Feng, Zhiqiang Fan, Sai Chen

**Affiliations:** 1 School of Electronic and Information Engineering, Beihang University, Beijing 100191, China; 2 School of Integrated Circuit Science and Engineering, Beihang University, Beijing 100191, China; 3 State Key Laboratory of Silicon and Advanced Semiconductor Materials, Zhejiang University, Hangzhou 310027, China

**Keywords:** NH_3_, MOFs, rGO, silicon micropillar array, gas sensor

## Abstract

This study develops high-performance ammonia sensors based on composites of metal-organic frameworks (MOF-808 and MOF-818) with reduced graphene oxide (rGO). Two sensor architectures were fabricated: interdigital electrodes and silicon micropillar arrays. The MOF-808/rGO composite demonstrated superior sensing performance for 40 ppm NH_3_ at room temperature, with faster response kinetics and higher sensitivity compared to pristine rGO and MOF-818/rGO. Silicon micropillar array sensors showed enhanced performance through optimized periodic arrangements, while oxygen plasma surface modification improved both sensor types. Comprehensive testing confirmed that the MOF-808/rGO sensor maintains reliable NH_3_ detection at concentrations as low as 5 ppm under high humidity conditions, exhibiting excellent stability and selectivity. These findings provide valuable insights for developing advanced gas sensors for environmental monitoring applications.

## 1. Introduction

Ammonia (NH_3_) is colorless at standard temperature and pressure, holding irreplaceable application value in agricultural production, chemical manufacturing, food processing, and other fields [1,2,3]. However, even minor leaks of this gas can constitute serious threats to the ecological environment, human health, and production safety. Research indicates that in flammable and explosive laboratory or industrial production environments with high safety requirements, even small ammonia emissions can severely endanger the lives of laboratory personnel and cause significant harm to the internal environment [4]. Particularly in long-term enclosed environments like industrial confined spaces and aerospace vehicles, developing high-performance, long-term stable room-temperature sensors for detecting trace ammonia concentrations demands greater sophistication. Consequently, creating low-concentration VOCs gas sensors capable of rapid, precise monitoring holds significant importance.

With developments in micro-nano fabrication technologies, silicon-based room-temperature gas sensors are continuously progressing toward miniaturization and integration. The most critical component determining the performance of these room-temperature sensors is the gas-sensitive sensing material. Research indicates that the most commonly used gas-sensitive materials currently are carbon-based nanomaterials and two-dimensional organic materials. These materials provide key support for breakthroughs in sensor performance due to their unique physicochemical properties [5,6,7,8]. For example, carbon nanotubes and reduced graphene oxide (rGO) possess larger specific surface areas, offering more active sites and accelerating electron migration rates, thereby significantly enhancing gas adsorption capacity. However, the response speed of single carbon-based materials is not ideal [9].

Recent advancements in gas sensing have demonstrated that integrating diverse material systems and constructing novel architectures can significantly overcome these limitations. Particularly, metal oxide semiconductor (MOS)-based gas sensors have achieved remarkable progress through microstructural design, defect engineering, and the construction of heterojunctions. Inspired by these structural and heterojunction-engineering strategies, research indicates that the blending of carbon-based materials with metal oxides, polymers, or porous frameworks can simultaneously enhance sensor selectivity, stability, and the response speed [10,11]. For example, S. Tohidi et al. employed a hydrothermal method to fabricate a novel high-performance chemoresistive gas sensor using a three-dimensional reduced graphene oxide/polyaniline (3DrGO/PANI) composite. They observed that the incorporation of PANA-NWs substantially improved both the response value and response speed of the gas sensor [12].

Among these, metal-organic frameworks (MOFs), as a type of porous material, also demonstrate immense potential. They possess outstanding advantages such as tunable pore size, ultra-large specific surface area, abundant active sites, and strong structural designability. Existing research indicates that MOFs exhibit excellent performance in gas storage and separation [13,14], carbon capture [15,16], hazardous substance monitoring [17,18], and heterogeneous catalysis [19,20]. In gas sensor fabrication, MOFs and their derivatives exhibit unique gas-sensing advantages. Xiao et al. reported a synthesis method for a CuO/ZnO composite derived from ZIF-8: first, using copper nitrate and zinc nitrate as metal sources, a suitable organic ligand was selected to synthesize a Cu-Zn bimetallic MOF precursor via a solvothermal process. Subsequently, high-temperature calcination ultimately formed a CuO/ZnO composite hollow nanomaterial that retained the cage-like structure of the MOF precursor. This material was applied to hydrogen sulfide (H_2_S) gas sensing. Experiments revealed its exceptional performance—including ultra-high sensitivity, rapid response, recovery, excellent selectivity, and good stability [21]. Begi et al. synthesized an indium oxide-modified cobalt tetroxide (In_2_O_3_-modified Co_3_O_4_) flower-like composite via a two-step process (combining hydrothermal and thermal treatment). Researchers further applied the synthesized In_2_O_3_/Co_3_O_4_ composite for ammonia (NH_3_) sensing. Results demonstrated a limit of detection (LOD) of 500 ppb at 250 °C, indicating excellent sensing performance [22].

Currently, MOFs have demonstrated advantages in gas sensor fabrication, particularly zirconium-based MOFs, which exhibit exceptional chemical and thermal stability [23]. This stems from the high charge density and strong coordination ability of zirconium ions (Zr^4+^). The Zr-O bonds formed with organic ligands (such as terephthalic acid) possess significantly higher bond energies than other chemical bonds like Zn-O or Cu-O bonds, endowing the framework with exceptional rigidity. This robust framework structure prevents structural deformation during adsorption–desorption cycles [24,25].

On the basis of these developments, this study employs MOF materials (MOF-808, MOF-818) combined with rGO as gas adsorbent materials. These composites were spray-coated onto silicon substrates using a spray gun to create MOF/rGO gas sensors. The sensing performance of the MOF/rGO gas sensor for ammonia (including response time, recovery time, concentration gradient, and repeatability) was systematically investigated. Oxygen plasma modification was applied to explore its impact on sensor performance. By introducing a silicon micropillar array as the sensor substrate and optimizing the silicon micropillar array through geometric arrangement control, the sensing performance of the array for NH_3_ at room temperature was systematically investigated, providing a novel approach for designing high-performance gas sensors.

## 2. Materials and Methods

### 2.1. Synthesis of MOF Materials

**Figure 1:**
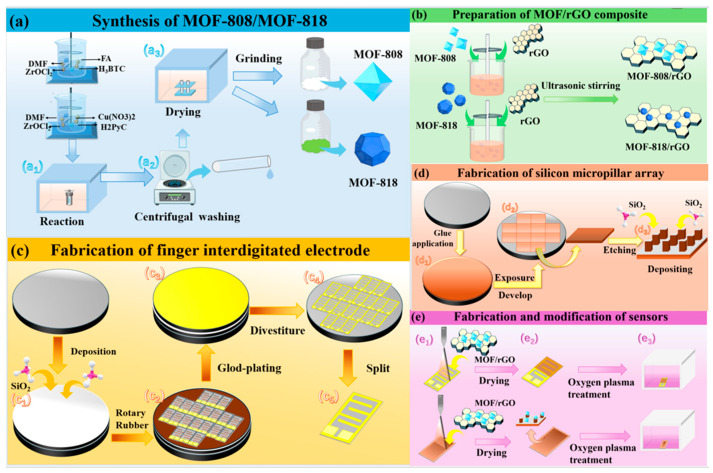
(**a**) Synthesis of MOF-808 and MOF-818 (a_1_–a_3_: solvothermal reaction, centrifugal washing, and drying/grinding, respectively); (**b**) Preparation of MOF/rGO composite; (**c**) Fabrication of finger interdigitated electrode (c_1_–c_5_: SiO_2_ deposition, spin coating of photoresist, gold deposition, lift-off process, and wafer splitting); (**d**) Fabrication of the silicon micropillar array (d_1_–d_3_: photoresist application, UV exposure/development, and silicon etching SiO_2_ deposition); (**e**) Fabrication and modification of sensors(e_1_–e_3_: spraying of sensing materials, drying, and oxygen plasma treatment).

The preparation process for MOF-808 is illustrated in Figure 1a. First, 1.94 g zirconium oxychloride octahydrate (ZrOCl_2_·8H_2_O), 0.42 g trimellitic acid (H_3_BTC) and 50 mL formic acid (FA) were dissolved in 50 mL N,N-dimethylformamide (DMF), followed by ultrasonic treatment for 30 min to ensure complete dissolution of the substances. Then, the mixture was transferred to a reaction kettle and reacted at 120 °C for 21 h (as shown in Figure 1(a_1_)). After reaction, centrifugation yielded a white precipitate. Then, the product was washed three times with DMF and methanol (as shown in Figure 1(a_2_)) to remove residual impurities. Finally, the precipitate was vacuum-dried at 120 °C for 8 h (as shown in Figure 1(a_3_)), and a white powder was obtained after grinding. The preparation of MOF-818 follows this procedure: 85 mg zirconium oxychloride octahydrate (ZrOCl_2_·8H_2_O), 62 mg copper nitrate (Cu(NO_3_)_2_·3H_2_O), and 65 mg 1H-pyrazole-4-carboxylic acid (H_2_PyC) were dissolved in 20 mL DMF. Ultrasonic dispersion for 5 min was followed by the addition of 240 μL trifluoroacetic acid. The mixture was reacted at 100 °C. After 10 h, another batch of copper nitrate (62 mg) was added to the solution, and the reaction was continued at 100 °C for an additional 10 h (as shown in Figure 1(a_1_)). The green crystals were collected by centrifugation and washed three times with DMF and acetone to remove impurities (as shown in Figure 1(a_2_)), then dried at 60 °C for 12 h (as shown in Figure 1(a_3_)). After grinding, a green powder was obtained.

### 2.2. Synthesis of MOF/rGO Composites

As shown in Figure 1b, the preparation process for the MOF/rGO composite materials (MOF-818/rGO and MOF-808/rGO) is as follows: 1 mL of 0.45 wt% rGO solution was mixed with 9 mL of deionized water in a beaker, and ultrasonic treatment was performed for 20 min at room temperature to ensure the rGO was fully dispersed, obtaining a uniform and stable 0.045 wt% rGO aqueous dispersion. Then, 50 mg of MOF material (MOF-818 or MOF-808) was added to the rGO aqueous dispersion (containing 4.5 mg of rGO). The MOF powder was initially dispersed by magnetic stirring for 10 min at room temperature, followed by another 20 min of ultrasonic treatment to ensure the MOF material was uniformly dispersed. This yielded the final MOF-818/rGO or MOF-808/rGO composite dispersion with a MOF concentration of 5 mg/mL and an rGO concentration of 0.45 mg/mL, corresponding to a constant MOF-to-rGO mixing weight ratio of 100:9.

### 2.3. Fabrication of Silicon-Based Finger Interdigitated Electrode and the Silicon Micropillar Array

As shown in Figure 1c, the fabrication process for the silicon-based finger interdigitated electrode is as follows: First, the silicon wafer was preprocessed (plasma cleaning), then cleaned with acetone, dried with nitrogen, and baked on a hot plate at 200 °C. Next, a silicon dioxide insulating layer with a thickness of approximately 400 nm was deposited on the silicon wafer using a PECVD (Plasma-Enhanced Chemical Vapor Deposition) device (Leuven Instruments, Xuzhou, China) (as shown in Figure 1(c_1_)). After that, the silicon wafer was placed in a spin coater, and S-1818 photoresist was spin-coated at a speed of 4000 rpm, followed by baking at 95 °C for 1 min to form a uniform and dense coating on the surface of the silicon wafer. Then, the silicon wafer coated with photoresist was exposed to ultraviolet light for 16 s using a mask with a preset pattern. After exposure, it was developed with a developer for 40–50 s, then cleaned and dried to form a photoresist mask with the target electrode pattern on its surface (as shown in Figure 1(c_2_)). Subsequently, double-layer metal films consisting of 40 nm titanium and 40 nm gold was deposited on the silicon wafer at 50 °C as electrodes (as shown in Figure 1(c_3_)), and then the wafer was cleaned with acetone and ethanol in sequence to remove residual photoresist and metal on the surface (as shown in Figure 1(c_4_)). Finally, the wafer was cut into unit structures of 1 cm × 0.5 cm (as shown in Figure 1(c_5_)). The fabrication process for the silicon micropillar array substrate is illustrated in Figure 1d. First, ma-N2403 photoresist was spin-coated using a spin coater at a speed of 4000 rpm and baked at 90 °C for 1 min to form a uniform and dense coating on the silicon wafer (as shown in Figure 1(d_1_)). Then, a dot-matrix photolithography mask was designed using L-Edit software (16.3), followed by exposure using a SUSS lithography machine (SUSS MicroTec SE, Garching, Germany) with the following exposure parameters: beam current 1.3–1.7 nA, dose 100 C/cm^2^. After exposure, the wafer was dried with nitrogen (as shown in Figure 1(d_2_)). Next, the wafer was etched using a deep silicon etcher to a depth of approximately 10 μm, forming silicon column structures. Subsequently, the residual photoresist on the wafer surface was removed by ultrasonic cleaning with acetone, followed by cleaning with ethanol and drying. Then, a 200 nm thick silicon dioxide insulating layer was deposited on the wafer using a PECVD device (as shown in Figure 1(d_3_)). Finally, the wafer was cut into samples of 1 cm × 0.5 cm, and tin was soldered diagonally on the sample to produce conductive contacts for subsequent measurements.

### 2.4. Fabrication and Modification of Silicon-Based Gas Sensors

As shown in Figure 1e, 0.5 mL of the MOF/rGO composite material was loaded into a spray gun and sprayed onto the surfaces of silicon-based interdigitated electrodes with different electrode widths and silicon micropillar array substrates with different periods (varying inter-micropillar spacings). During spraying, a uniform and appropriate speed was maintained to avoid forming obvious droplets on the substrate surface. During the spraying process, the MOF/rGO composite material adhered to the substrate in the form of an aerosol (as shown in Figure 1(e_1_)). Finally, the samples were placed in a vacuum oven and dried at 80 °C for 2 h to obtain the gas sensors (as shown in Figure 1(e_2_)). The sensors were placed in a plasma surface treatment unit for surface modification via oxygen plasma bombardment (as shown in Figure 1(e_3_)). The plasma treatment conditions were as follows: first, the chamber was evacuated, then 50 sccm of oxygen and 20 sccm of argon were injected into the chamber, and the discharge was maintained at a power of 200 W for 30 s and 120 s to obtain two types of gas sensors with different modification degrees.

### 2.5. Gas Sensing Performance Test Method

The gas-sensing performance of the sensors was evaluated using a gas sensor testing system (JF02F, Guiyang Jinfeng, Guiyang, China). As illustrated in the schematic diagram of the experimental setup (Figure S1), the test chamber had a volume of approximately 77 mL. To assess the gas-sensing properties of the MOF/rGO sensors, the resistance variation curves of the sensors exposed to 5–50 ppm NH_3_ were recorded using a dynamic testing method under ambient conditions of 30 ± 2 °C and 40% relative humidity (RH). The gas flow was strictly regulated using high-precision mass flow controllers (MFCs), ensuring a constant total gas flow rate of 125 sccm throughout all tests. Synthetic air was utilized as both the background and carrier gas for the measurements. All sample gases used in the experiments (100 ppm NH_3_ balanced with N_2_, 500 ppm NO_2_ balanced with N_2_, 100 ppm C_2_H_6_O balanced with N_2_, and 99.6 ppm H_2_S balanced with N_2_) were purchased as commercial standard gas cylinders from Beijing Huatong Jingke Gas Chemical Co., Ltd. (Beijing, China). According to the manufacturer’s certification, these standard gases were strictly prepared using the gravimetric method (in accordance with the GB/T 5274-2008 standard [26]) from gas components of precisely known purities. Therefore, 100% pure target gases or liquid sources (e.g., for the C_2_H_6_O VOC sample) were not utilized in our laboratory. All original source gases from these cylinders were completely dry. Testing concentrations of NH_3_ (5–50 ppm) were dynamically calibrated and generated by proportionally mixing the 100 ppm NH_3_ standard source gas (balanced with N_2_) with the dry synthetic air carrier gas using the MFCs. For tests requiring specific humidity levels, the relative humidity was regulated by proportionally blending the dry gas stream with a humidified gas stream generated by flowing synthetic air through deionized water, and the gas humidity was monitored in real time by a humidity sensor inside the chamber.

This study focused on investigating the key sensing performance metrics of the sensor: response time (*t_resp_*), recovery time (*t_rec_*), repeatability, and response to NH_3_ with varying concentrations. The response time (*t_resp_*) is defined as the time required for the sensor to achieve 90% of the total resistance change upon the introduction of the target gas. The recovery time (*t_rec_*) refers to the duration needed for the sensor resistance to return to 90% of its initial baseline value after the target gas supply is terminated.

Response(%) is defined by Equation (1):

(1) $$\mathrm{Response}(\%)\;=\;\frac{\left|R_{0}-R\right|}{R_{0}}\times 100\%$$

where *R* is the real-time resistance of the gas sensor, and *R*_0_ is the resistance of the sensor at a stable state after exposure to the background gas. The same definition of Response(%) was applied for both reducing and oxidizing gases.

Furthermore, in the data processing stage, the *R*/*R*_0_ response was defined, where *R* denotes the real-time resistance of the sensor, and *R*_0_ is the resistance of the sensor at a stable state after exposure to the background gas.

## 3. Results and Discussion

### 3.1. Characterization of MOFs and MOF/rGO

**Figure 2:**
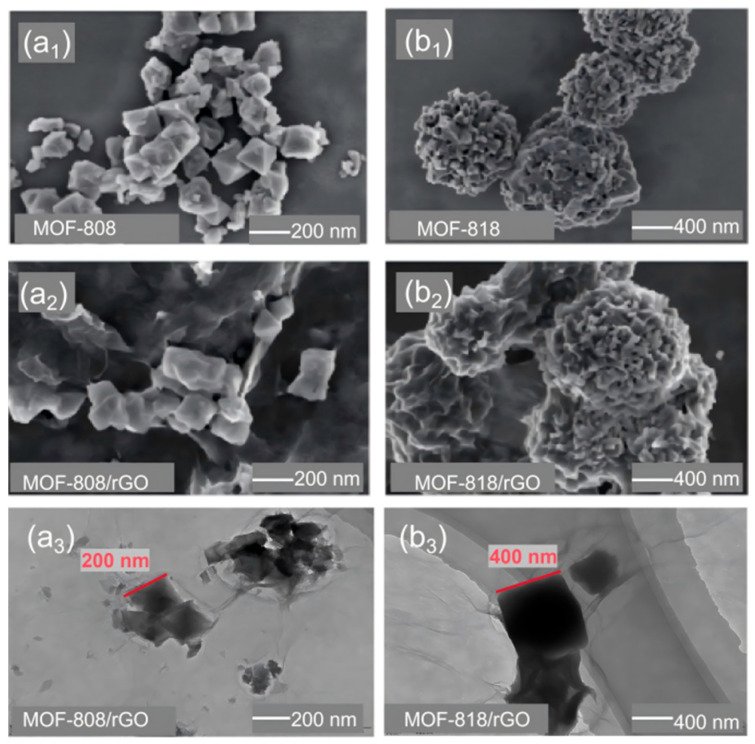
SEM images show (**a_1_**) MOF-808; (**a_2_**) MOF-818; (**b_1_**) MOF-808/rGO; (**b_2_**) MOF-818/rGO; TEM image of (**a_3_**) MOF-808/rGO; (**b_3_**) MOF-818/rGO.

As shown in Figure 2(a_1_,a_2_,b_1_,b_2_), surface morphology characterization of MOF-808, MOF-818, and MOF/rGO samples was performed by scanning electron microscopy (SEM). Figure 2(a_1_) illustrates that the synthesized MOF-808 sample possesses a regular octahedral crystal structure, with well-dispersed particles and only slight localized agglomeration. Individual crystals exhibit distinct boundaries, with an average grain size of approximately 200 nm. Figure 2(b_1_) reveals that the synthesized MOF-818 sample exhibits a multiscale agglomerated morphology, specifically a “spherulite-like agglomerate structure” formed by the stacking of numerous minute octahedral crystals, with the overall agglomerate size being approximately 600 nm. Figure 2(a_2_) and Figure 2(b_2_) display the surface morphology of MOF/rGO samples synthesized by mixing MOF-808 and MOF-818 with rGO, respectively. Compared to pure MOF materials, the introduction of rGO does not alter the fundamental morphology of the MOFs itself. MOF crystals are dispersed and attached to the surface and layered structure of rGO, achieving a tight composite between MOFs and rGO. Transmission electron microscopy (TEM) analysis of MOF-808/rGO and MOF-818/rGO reveals (as shown in Figure 2(a_3_,b_3_)) MOF crystalline particles consistent with the SEM morphology, alongside rGO’s flaky substrate (light, transparent, wrinkled layered structures). MOF crystals are dispersed and attached to the surface of rGO sheets, with a clear interface between the two. The MOF crystals show no significant agglomeration, indicating good dispersion. The EDS spectra of the MOF/rGO composites are shown in Figure S2. O and C elements are detected across both MOFs and rGO phases, while Zr is uniformly distributed only within the MOF-818 aggregates and the octahedral crystal domains of MOF-808. This further confirms that the MOFs retain their structural integrity after blending with rGO.

Furthermore, XRD analysis was conducted to evaluate the crystal structure of the composite. As shown in Figure S3, the XRD pattern of the MOF-808/rGO composite perfectly retains the sharp and intense characteristic peaks of pristine MOF-808. Notably, no significant peak shifts or Scherrer broadening effects were observed after the introduction of rGO. This observation is highly consistent with our post-synthetic room-temperature ultrasonic blending strategy. Since the highly crystalline MOF-808 framework was already fully formed prior to the mixing process, the rGO nanosheets primarily adhered to the external surfaces of the MOF-808 crystals without incorporating into the metal-organic lattice or restricting its initial crystal growth. These results further demonstrate that the structural integrity and high crystallinity of MOF-808 are well-preserved during the composite formation process.

The BET specific surface area and pore structure data of MOF-808 and MOF-818 are presented in Figure S4 and Table 1. Comparative analysis reveals that the average pore diameter of MOF-808 is about 3.05 nm, which is greater than 2 nm, classifying it as a mesoporous material; in contrast, the average pore diameter of MOF-818 is about 1.84 nm, less than 2 nm, thus belonging to the microporous category. Moreover, MOF-808 exhibits higher specific surface area and total pore volume than MOF-818, indicating that MOF-808 possesses a more abundant pore channel structure. A larger number of pore channels with suitable diameters can provide more active sites, which is conducive to the adsorption and capture of small molecules such as NH_3_. Given these characteristics, do MOF materials hold inherent advantages for NH_3_ detection? To address this question, the sensing performance of NH_3_ sensors coated with MOF/rGO composites was further investigated.

### 3.2. Gas Sensing Performance

To evaluate the gas-sensing performance of the MOF/rGO composite sensors, the resistance variation curves of the sensor for 5–50 ppm NH_3_ were measured via a dynamic testing method under ambient conditions of 30 ± 2 °C and RH 40%.

#### 3.2.1. Gas-Sensing Performance of Interdigitated Electrode Gas Sensor

**Figure 3:**
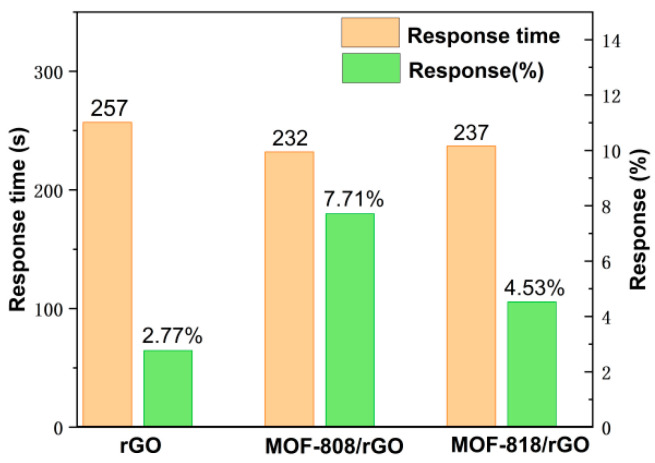
Comparison of responses of rGO and MOF/rGO sensors to 40 ppm NH_3_.

Three interdigitated electrodes with widths of 152.2 μm, 202.2 μm, and 253.1 μm were used, and the same amount of rGO solution was spray-coated on their surfaces. The response–recovery curves for 40 ppm NH_3_ are shown in Figure S5(a1–a3). Subsequently, the MOF-808/rGO and MOF-818/rGO composite dispersions were coated onto identical electrodes for testing. Curves for MOF-818/rGO and MOF-808/rGO are presented in Figure S5(b_1_–b_3_) and (c_1_–c_3_), respectively. All sensors showed resistance changes upon NH_3_ exposure. rGO and MOF-808/rGO sensors exhibited a resistance decrease, while MOF-818/rGO showed a resistance increase. Benefiting from abundant active sites, MOF/rGO sensors showed stronger NH_3_ adsorption, shorter response time, and higher response than pure rGO sensors at the same electrode width. For identical sensing materials, the 253.1 μm electrode gave the fastest response. The MOF-808/rGO sensor with this width achieved a response time of 197 s, improved by about 21.2%. As shown in Figure 3, average response times were 257 s for rGO, 237 s for MOF-818/rGO, and 232 s for MOF-808/rGO. The average Response(%) values were 2.77% for rGO, 4.53% for MOF-818/rGO, and up to 7.71% for MOF-808/rGO. The greatly enhanced response is attributed to the introduction of MOFs, which significantly improve the NH_3_ adsorption capacity of the composites.

Figure S6(a_1_,b_1_) present the repeatability tests of the MOF/rGO sensors exposed to 40 ppm NH_3_. As can be seen from the figures, the Response(%) value, response time, and recovery time of the MOF/rGO sensors for NH_3_ remained stable over three consecutive test cycles, indicating that the MOF/rGO sensors possessed excellent repeatability. In addition, Figure S6(a_2_,b_2_) depict the resistance curves of the samples in the NH_3_ concentration range of 10–50 ppm. It was found that the sensors still exhibited detectable responses to NH_3_ even with decreasing NH_3_ concentrations, but their sensing performance gradually deteriorated, characterized by a reduction in Response(%) value and an increase in response time.

Based on response–recovery curves and Figure 3 data, the sensing performances of MOF-808/rGO and MOF-818/rGO sensors were compared. The MOF-808/rGO sensor exhibited a 3.18% higher average Response(%) to 40 ppm NH_3_, with a minimum response time of 197 s versus 220 s for MOF-818/rGO. Its recovery behavior was also superior (Figure S5). These results confirm that MOF-808 is more favorable for NH_3_ sensing, so MOF-808/rGO was chosen for further investigations.

To further enhance the response performance of the gas sensor, oxygen plasma treatment technology was employed for the surface modification of MOF-808/rGO devices. The fabricated gas sensors were placed in a plasma processor, with an oxygen flow rate of 50 sccm and an argon flow rate of 20 sccm introduced into the chamber. After glow discharge was initiated to generate oxygen plasma, the sensors were treated for durations of 30 s and 120 s, respectively, yielding two types of sensors with different modification degrees. Subsequently, the gas sensors were stored for 24 h to stabilize their device states prior to performance testing. This aging process ensured that the device resistance and surface properties reached a steady state, eliminating the impact of initial relaxation effects induced by plasma treatment on the test results and thus guaranteeing the reliability and repeatability of subsequent gas-sensing performance evaluations.

**Figure 4:**
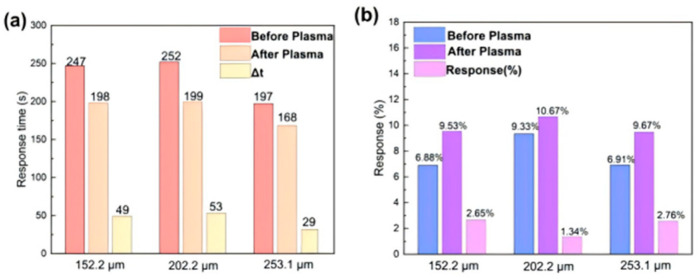
(**a**) Comparison of response time; (**b**) Comparison of Response(%) of interdigitated electrode-based MOF-808/rGO sensors exposed to 40 ppm NH_3_ before and after 120 s of plasma treatment.

Figure S7 presents the response–recovery curves of plasma-treated MOF-808/rGO sensors exposed to 40 ppm NH_3_. Plasma treatment significantly increased the initial resistance, due to disrupted surface structure, introducing defects and oxygen-containing groups that changed the conductivity. Despite this, the resistance variation trend remained unchanged. Plasma treatment greatly shortened response time and obviously improved response, owing to more surface active sites that enhance NH_3_ adsorption and charge transfer. As shown in Figure 4, after 120 s treatment, the average response time of three electrodes decreased by 18.8% to 188.3 s. The 253.1 μm device achieved the fastest response of 168 s (reduced by 29 s). The Response(%) value increased by 1.64–2.76%, with the average reaching 12.12%, a 57.3% enhancement.

Through the investigation of interdigitated electrode sensors and screening of sensing materials, an NH_3_ gas sensor based on the MOF-808/rGO composite was fabricated. This sensor exhibited excellent gas-sensing performance toward 40 ppm NH_3_ under ambient temperature conditions. In addition to the gas-sensitive materials, the substrate structure of the sensor is also a key factor affecting the sensing performance. In recent years, researchers have found that 3D substrate structures can provide gas-sensitive materials with a larger specific surface area and more efficient gas diffusion pathways and electron transport channels, thereby enhancing the performance of gas sensors [27,28,29]. Therefore, to further improve the response capability of the MOF-808/rGO sensor toward NH_3_, silicon micropillar arrays with square arrangements and different periods were prepared as the sensor substrate structures, and their morphology and gas-sensing performance were systematically investigated.

#### 3.2.2. Gas-Sensing Performance of the Silicon Micropillar Array Sensors

**Figure 5:**
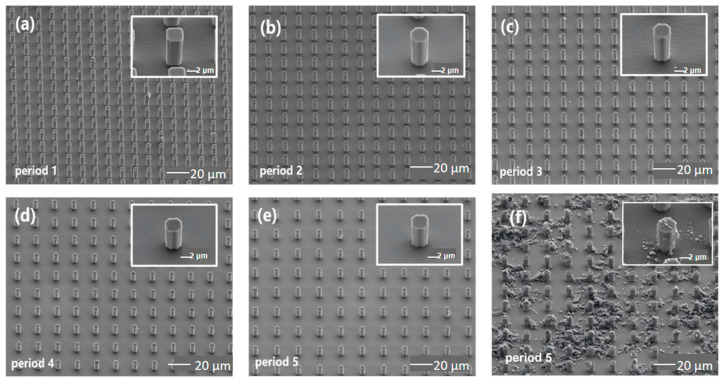
(**a**–**e**) SEM images of the silicon micropillar array at a 45° tilt angle ((**a**–**e**) correspond to samples period-1, period-2, period-3, period-4, period-5 in sequence). Specifically, samples period-1 to period-5 featured square lattices with a side length of 5 μm, The inter-micropillar spacing of samples period-1 to period-5 increased sequentially as follows: 8.2 μm, 10.2 μm, 12.2 μm, 14.2 μm, 16.2 μm). (**f**) SEM images of the silicon micropillar array coated with MOF-808/rGO at a 45° tilt angle (period-5).

Morphological characterization of the square-patterned silicon micropillar arrays was carried out, with SEM results shown in Figure 5. Figure 5a–e present 45° tilted SEM images of period-1 to period-5 samples. All micropillars had a side length of 5 μm, while the inter-pillar spacing gradually increased from 8.2 μm to 16.2 μm. The arrays showed good verticality and uniformity, and the magnified inset (2 μm scale) revealed slight passivation at the square corners but no overall distortion. All samples exhibited a consistent etching depth of 10 μm. The MOF-808/rGO composite was prepared in the same ratio used for interdigitated electrode sensors, then uniformly spray-coated onto different micropillar arrays and dried to form gas sensors. SEM characterization (Figure 5f, 45° tilt) showed that MOF-808/rGO was successfully deposited on the tops, sidewalls, and bottoms of the micropillars. The planar top and bottom surfaces were covered with a relatively thick, uniform coating. By contrast, coating coverage on the vertical sidewalls was lower and less uniform, showing a sparse upper and dense lower distribution. This inhomogeneity is attributed to the gravitational settling of MOF-808 crystals and the adhesion of rGO during spraying. Despite the non-uniform coating, the 3D micropillar array provides a much larger specific surface area than planar substrates, enhancing gas adsorption and active sites, which effectively improves sensor response performance.

**Figure 6:**
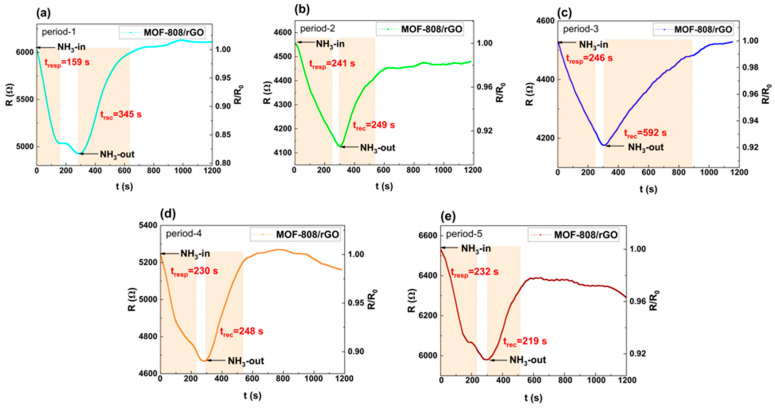
Response–recovery curves of the silicon micropillar array MOF-808/rGO sensors exposed to 40 ppm NH_3_ with different arrangement periods (inter-micropillar spacings): (**a**) period-1 (8.2 μm); (**b**) period-2 (10.2 μm); (**c**) period-3 (12.2 μm); (**d**) period-4 (14.2 μm); and (**e**) period-5 (16.2 μm).

**Figure 7:**
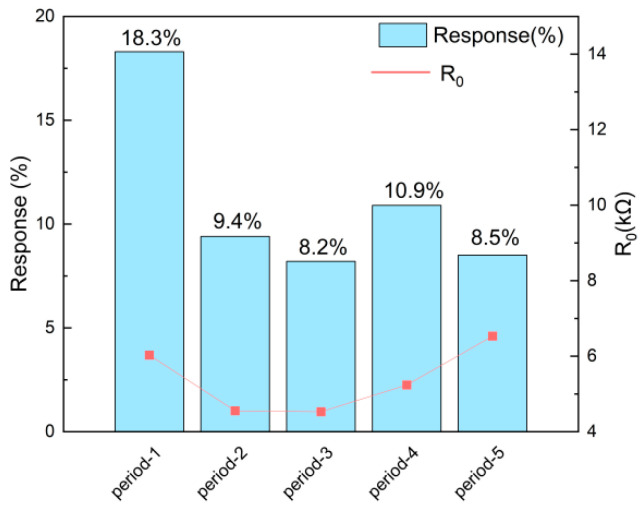
Diagram comparing initial resistance and Response(%) of period-1 to period-5 sensors.

To investigate the influence of arrangement periods on sensing performance, period-1 to period-5 silicon micropillar sensors were tested with 40 ppm NH_3_ at 30 ± 2 °C and 40% RH. Figure 6 shows their response–recovery curves. Upon exposure to NH_3_, all sensors showed decreasing resistance within 300 s and gradually reached adsorption equilibrium. After introducing air, the resistance recovered to the initial level. Figure 7 compares the initial resistance and Response(%) of period-1 to period-5 sensors. With the same MOF-808/rGO coating, the initial resistance of micropillar-based sensors was around 6 kΩ, much lower than that of interdigitated electrode sensors.

In addition, as can be seen from the data in Figure 6 and Figure 7, the sensing response capabilities of the sensors vary significantly with different periods. Among them, the period-1 sensor (with a micropillar side length of 5 μm and an inter-micropillar spacing of 8.1 μm) exhibits significantly faster response speeds and higher Response(%) values on exposure to 40 ppm NH_3_ than the period-2 to period-5 sensors, with a response time of 159 s, and Response(%) value of 18.3%. This phenomenon arises because the different inter-micropillar spacings affect the flow velocity of NH_3_ between the micropillars as well as the specific surface area of the silicon micropillar array, thereby influencing the binding rate between gas molecules and the gas-sensitive material, as well as the number of adsorption active sites. Overall, the period-1 sample demonstrates the fastest response speed and the highest Response(%).

Figure S8 shows the resistance variation curves of the five types of sensors on exposure to NH_3_ in the concentration range of 5–40 ppm, where (a)–(e) correspond to period-1 to period-5 in sequence. During the continuous test with concentration gradients, the resistance of all sensors exhibited a decreasing trend in response to NH_3_. Notably, all types of sensors also demonstrated favorable response and recovery capabilities even at a low concentration of 5 ppm. With the increase in NH_3_ concentration, the resistance variation of the gas sensors gradually increased.

To further improve the sensor performance, surface modification was performed on period-1 to period-5 sensors via oxygen plasma treatment technology. The fabricated gas sensors were placed in a plasma processor with an oxygen flow rate of 50 sccm and an argon flow rate of 20 sccm. After glow discharge was initiated to generate oxygen plasma, the sensors were treated continuously for 30 s and 120 s, yielding two types of sensors with different modification degrees. Subsequently, the gas sensors were stored for 24 h to stabilize their device states prior to performance testing. This step ensured that the device resistance and surface states reached a steady condition, eliminating the impact of initial relaxation effects after treatment on test results and thus guaranteeing the reliability and repeatability of subsequent gas-sensing performance evaluations.

**Figure 8:**
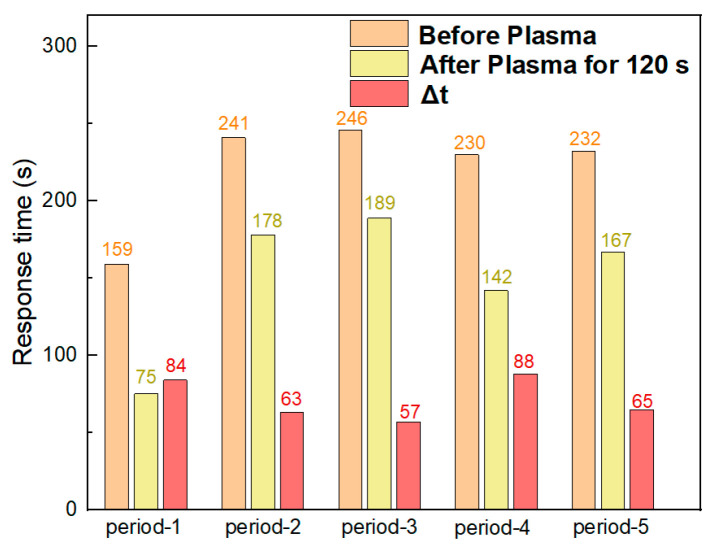
Comparison of response time of silicon micropillar array MOF-808/rGO sensors before and after 120 s plasma treatment.

The response–recovery curves of the plasma-treated gas sensors exposed to 40 ppm NH_3_ are presented in Figure S9. As can be seen from the figure, after NH_3_ injection, the resistance of period-1 to period-5 sensors all exhibited a decreasing trend; when air was introduced into the test chamber, the resistance recovered to the initial state, which was consistent with the resistance variation trend before plasma treatment. Table 2 records the changes in initial resistance values of the sensors before and after plasma treatment. It can be observed that the initial resistance of period-1 to period-5 sensors increased significantly after plasma treatment. This phenomenon indicates that for silicon micropillar array sensors, oxygen plasma bombardment still introduces new oxygen-containing functional groups into the materials, and these functional groups alter the electrical conductivity of the materials, resulting in an increase in resistance. A comparison of the sensing performance of the five types of sensors before and after plasma treatment reveals that the Response(%) values of the sensors basically decreased after plasma treatment, whereas the resistance variation values of the sensors increased. Notably, as shown in Figure 8, the response time of period-1 to period-5 sensors was shortened after plasma treatment compared with the untreated sensors. Among them, the response time of period-1 after 120 s of plasma treatment was only 75 s, which was reduced by 84 s compared with that before treatment. This result demonstrates that the surface defects and oxygen-containing functional groups introduced by oxygen plasma treatment increase the active sites for reactions between the gas-sensitive materials and NH_3_, effectively enhancing the adsorption capacity of the materials for NH_3_ and accelerating the process of the silicon micropillar array sensors reaching response equilibrium.

**Figure 9:**
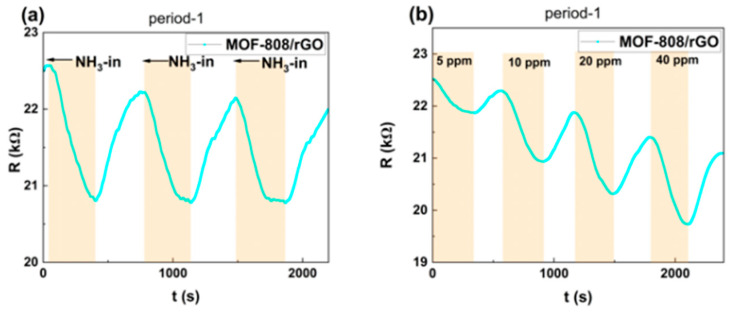
(**a**) Reproducibility test and (**b**) concentration gradient test of period-1 after 120 s plasma treatment.

Overall, sample period-1 still exhibited the optimal sensing performance after plasma treatment. Reproducibility tests and concentration gradient tests in the range of 5–40 ppm were conducted on period-1 after 120 s of plasma treatment, with the results presented in Figure 9. As shown in Figure 9a, the response time and response value of the period-1 sensor remain stable over three cycles, indicating that the plasma treatment does not compromise the stability of the sensor. As depicted in Figure 9b, the sensor still demonstrates a distinct response toward 5 ppm NH_3_, exhibiting excellent detection capability.

**Figure 10:**
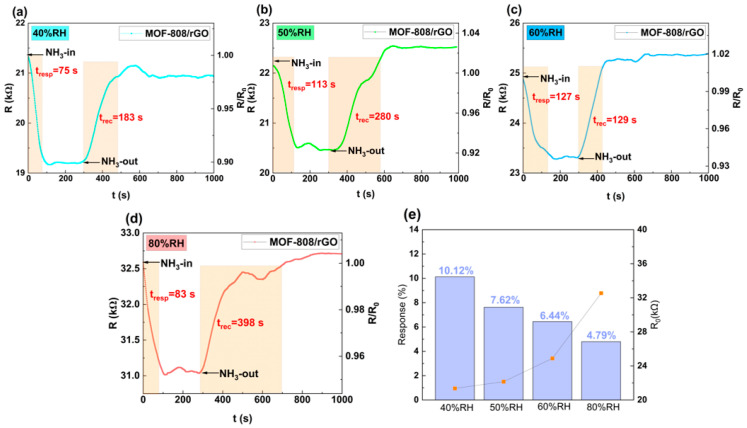
(**a**–**d**) Response–recovery curves of period-1 sensor after 120 s plasma treatment, on exposure to 40 ppm NH_3_ at RH of 40–80%; (**e**) Initial resistance and Response(%) of period-1 sensor under different relative humidity conditions.

Humidity significantly influences the performance of room-temperature gas sensors. The period-1 sensor with 120 s plasma treatment was tested at 40%, 50%, 60%, and 80% RH for its response to 40 ppm NH_3_, with response–recovery curves shown in Figure 10a–d. The sensor remained responsive over 40–80% RH. As shown in Figure 10e, initial resistance increased with humidity, reaching 32.54 kΩ at 80% RH (11.18 kΩ higher than at 40% RH). Water molecules adsorbed through hydrogen bonds hindered carrier transport and reduced carrier concentration, raising resistance. Within 40–60% RH, response time lengthened and response value declined with increasing humidity: at 60% RH, response time was 127 s and Response(%) was 6.44%, 52 s slower and 3.68% lower than at 40% RH, due to competitive adsorption between H_2_O and NH_3_. At 80% RH, Response(%) dropped further to 4.79%, but response time shortened to 83 s. Excess water occupied most active sites, weakening NH_3_ adsorption and accelerating resistance saturation.

**Figure 11:**
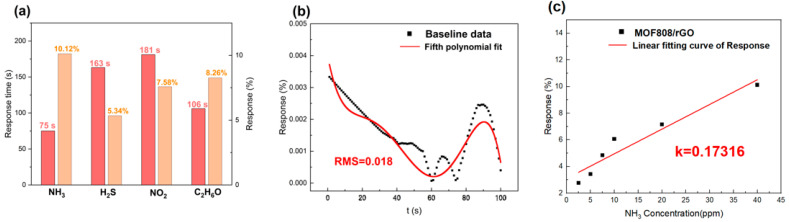
(**a**) Comparison of sensing performance of period-1 sensor exposed to 40 ppm NH_3_, H_2_S, NO_2_ and C_2_H_6_O; (**b**) Fifth-order polynomial linear fitting of baseline data of period-1 sensor; (**c**) Linear fitting between response value and NH_3_ concentration of period-1 sensor.

To evaluate the selectivity of the period-1 sensor, three gases—H_2_S, NO_2_, and C_2_H_6_O—were selected as the interfering gases, and the sensing performance of the sensor to these three gases was tested at a concentration of 40 ppm, with the results presented in Figure 11a. As can be seen from the figure, the response times of the period-1 sensor to 40 ppm H_2_S, NO_2_, and C_2_H_6_O were 163 s, 181 s, and 106 s, respectively, while the response time to 40 ppm NH_3_ was 75 s, which was reduced by 88 s, 106 s, and 31 s compared with the responses times to H_2_S, NO_2_, and C_2_H_6_O, respectively. The Response(%) values of the period-1 sensor to 40 ppm H_2_S, NO_2_, and C_2_H_6_O were 5.34%, 7.58%, and 8.26%, respectively, whereas the Response(%) to 40 ppm NH_3_ was 10.12%, which was 1.90, 1.34, and 1.23 times that of H_2_S, NO_2_, and C_2_H_6_O, respectively. The above results indicate that the sensor exhibits stronger response capability toward NH_3_ than toward H_2_S, NO_2_, and C_2_H_6_O, demonstrating that the period-1 sensor has high selectivity for NH_3_.

The limit of detection (LOD) is another critical performance indicator for gas sensors, and is defined by Equation (2):

(2) $$LoD=\frac{3RMS}{K}$$

Among these parameters, the root mean square (RMS) value is derived from the fifth-order polynomial linear fitting of the baseline (Figure 11b), with a calculated value of 0.018. The slope k is obtained via the linear fitting curve between the response value and NH_3_ concentration (Figure 11c), and its value is determined to be 0.17316. Using the RMS and k values, the theoretical limit of detection (LOD) of the period-1 sensor was calculated to be 0.312 ppm.

### 3.3. Gas Sensing Mechanism

The as-fabricated MOF-808/rGO sensor exhibits excellent NH_3_ sensing performance, which is attributed to the favorable synergistic effect between the porous structure of MOF-808 and the superior electrical conductivity of reduced graphene oxide (rGO). First and foremost, during the chemical reduction of graphene oxide (GO) to rGO, abundant structural defects (e.g., carbon vacancies and edge sites) and residual oxygen-containing functional groups (mainly hydroxyl and carboxyl groups) are well preserved. These structural and chemical characteristics endow rGO with outstanding gas molecular adsorption capability, thus rendering it with the core active sites for NH_3_ recognition and interaction [30]. Specifically, the oxygen atoms in hydroxyl groups possess high electronegativity, which induces the formation of an asymmetric electron density distribution within the hydroxyl groups and further makes the directly bonded hydrogen atoms carry weak positive charges. The positively charged hydrogen atoms can form stable O-H···N hydrogen bonds with the lone-pair electrons on the nitrogen atoms of NH_3_ molecules. Similarly, each carboxyl group contains two electronegative oxygen atoms, which further enhances the electron-withdrawing effect, leading to a significant electron density shift inside the functional groups and thereby making the hydrogen atoms in carboxyl groups effective hydrogen bond donors. Therefore, carboxyl groups can interact with NH_3_ molecules to form C=O···H-N hydrogen bonds, thus facilitating the adsorption of NH_3_ molecules on the rGO surface [30,31]. In addition, the van der Waals forces between the rGO surface and NH_3_ molecules further enhance the adsorption stability, providing a favorable microenvironment for the subsequent charge transfer process. Notably, the formation of hydrogen bonds significantly reduces the energy barrier for charge transfer at the rGO-NH_3_ interface. After being adsorbed on the rGO surface via hydrogen bonds, NH_3_ molecules will inject electrons into the rGO matrix [31]. Since the rGO used in this work is of n-type, the injection of electrons increases the carrier concentration in the rGO matrix. Macroscopically, this process is manifested as a significant decrease in the resistance of the rGO sensing layer.

To fundamentally understand this resistance change, it is essential to analyze the energy-band structure and charge-transfer dynamics at the metal–semiconductor interface (as illustrated in Figure S10). In our composite, reduced graphene oxide (rGO) exhibits n-type semiconductor behavior. When electron-donating NH_3_ molecules are trapped by the MOF-808/rGO interface, they inject electrons directly into the conduction band of the rGO matrix. This massive injection of charge carriers increases the electron concentration, causing the Fermi level (E_F_) of rGO to shift upward, closer to its conduction band minimum (E_C_). Furthermore, the electrical signal of the micropillar sensor is extracted via Tin (Sn) contacts, which inherently forms a Schottky barrier at the interface between the Sn electrode and the n-type rGO. Upon NH_3_ exposure, the gas molecules concurrently adsorb near the Sn/rGO interface, modulating and locally reducing the work function of the Sn metal. This reduction in the metal work function, combined with the upward shift of the rGO Fermi level, significantly narrows and lowers the effective Schottky barrier height. This lowered barrier exponentially facilitates electron injection and extraction via thermionic emission and tunneling. Meanwhile, the 3D silicon micropillar scaffold physically prevents the restacking and agglomeration of rGO sheets. This structural preservation ensures that the injected electrons have a continuous, unhindered 3D percolation network with high carrier mobility, ultimately resulting in the rapid and sharp decrease in macroscopic resistance observed in the device.

**Figure 12:**
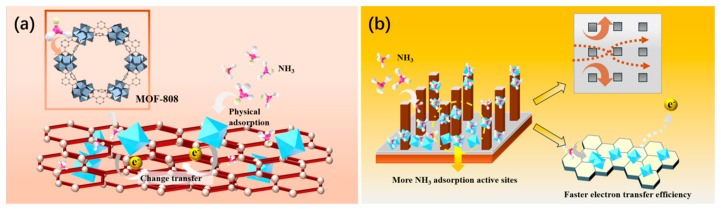
(**a**) Schematic illustration of the NH_3_ sensing mechanism of MOF-808/rGO; (**b**) Schematic illustration of the sensing mechanism of the silicon micropillar array.

Secondly, MOF-808 possesses an ultrahigh specific surface area and a porous structure with a pore size larger than the kinetic diameter of NH_3_ (0.326 nm) [32,33]. As illustrated in Figure 12a, MOF-808 can rapidly capture NH_3_ molecules via physical adsorption driven by van der Waals forces, thus achieving efficient enrichment of the target gas. Beyond this physical enrichment, the hydrogen bonding interactions between NH_3_ and the MOF-808 framework plays a pivotal role in enhancing the NH_3_ adsorption capacity. Our prior investigation has systematically elucidated that the hydroxyl groups and aqua ligands at the Zr_6_ nodes, specifically the μ3-OH and t-OH/OH_2_ species, serve as key hydrogen bond donors for NH_3_ molecules [34]. In addition, the porous structure of MOF-808 ensures the rapid diffusion of NH_3_ molecules even at low concentrations, endowing the sensor with a faster response rate. In the as-prepared MOF-808/rGO composite, MOF-808 mainly serves to enhance the NH_3_ adsorption capacity, while rGO acts as a conductive material to provide electron transport pathways. The synergistic effect between MOF-808 and rGO effectively improves the overall sensing performance of the sensor.

**Figure 13:**
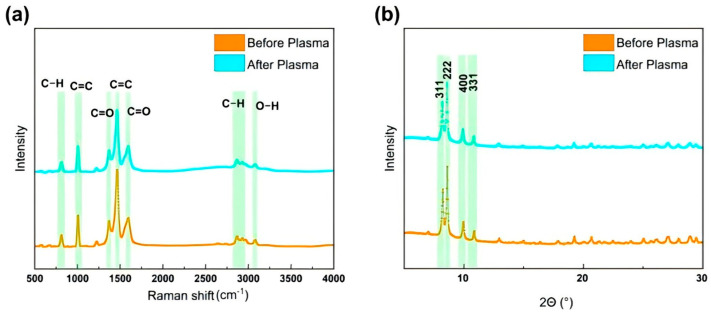
(**a**) Raman spectra of MOF-808 before and after plasma treatment; (**b**) XRD patterns of MOF-808 before and after plasma treatment.

Furthermore, plasma treatment introduces more surface defects and oxygen-containing functional groups [35], increasing active sites for NH_3_ adsorption and enhancing the adsorption capacity of the composite. Raman and XRD characterizations of MOF-808 before and after 120 s oxygen plasma treatment are shown in Figure 13. As seen in the Raman spectrum (Figure 13a), pristine MOF-808 exhibited strong, sharp characteristic peaks reflecting high crystallinity. After plasma treatment, overall Raman intensity decreased by an average of 42%, especially at high wavenumbers. The characteristic peak of C=O coordination vibration at 1500–1800 cm^−1^ weakened from 78 to 43 (45% attenuation) and broadened, suggesting partial desorption or oxidation of surface carboxyl groups and physical adsorption of new oxygen-containing species. Increased FWHM of all peaks confirmed the formation of abundant lattice defects and possible reduced grain size. No new peaks appeared, indicating no phase transition or impurity formation, only surface modification and local structural adjustment [36,37,38]. XRD results (Figure 13b) showed obvious attenuation of low-angle (5–15°) peaks, indicating damaged long-range crystalline order, while no peak shift implied unchanged bulk lattice parameters. Peak broadening further verified defect introduction, and no new or vanished peaks confirmed no phase transition. Overall, oxygen plasma treatment generates abundant surface defects and weak interactions with coordinatively unsaturated metal sites, causing temporary coordination or local oxidation without destroying the main framework or inducing phase transition [39].

While the Raman analysis (Figure 13) primarily confirms the structural stability of the MOF-808 framework during the modification process, the simultaneous surface evolution of the rGO matrix plays an equally critical role in the enhanced sensing mechanism. Due to signal overlapping, the intrinsic defect variations of rGO within the composite are challenging to isolate directly. However, the mechanism of plasma-induced defect generation in carbonaceous materials is well established. As comprehensively demonstrated in a recent study by Liu et al. [40], room-temperature plasma bombardment significantly elevates the defect density of rGO, evidenced by a marked increase in the Raman I_D_/I_G_ ratio (e.g., from 1.18 to 1.31). This signifies the generation of abundant structural defects, such as carbon vacancies and sp^3^ hybridized regions. In our MOF-808/rGO synergistic system, these plasma-induced defects on the rGO basal plane act as high-energy active sites. They not only provide highly reactive centers for the specific adsorption of NH_3_ molecules but also significantly lower the activation energy for the subsequent charge transfer. Consequently, the plasma-engineered defect density of rGO fundamentally accelerates the response kinetics and enhances the overall sensitivity of the sensor.

To further validate the surface evolution induced by the plasma treatment, high-resolution XPS O 1s spectra of the MOF-808/rGO composites before and after treatment were provided in the Supporting Information (Figure S11 and Table S1). The deconvolution results reveal that upon plasma treatment, the absolute peak area of lattice oxygen (O_L_) decreased, accompanied by an increase in the absolute area of oxygen vacancies (O_V_). This demonstrates that the plasma bombardment successfully breaks intrinsic M-O bonds, introducing structural defects on the micropillar surfaces. More notably, the chemisorbed oxygen species (O_C_) exhibited a massive enhancement, with its relative proportion increasing from 24.89% to 35.83%. These newly generated oxygen vacancies and abundant functional groups serve as highly active adsorption sites for NH_3_, fundamentally synergizing with the 3D scaffold to accelerate gas diffusion and charge transfer.

In this work, it was also found that the silicon micropillar array sensor exhibited superior sensing performance compared with the interdigital electrode sensor. As depicted in Figure 12b, this is because the silicon micropillar structure possesses abundant gas flow channels, which not only provide multi-directional and rapid diffusion pathways for NH_3_ molecules, reducing molecular transport resistance, but also enable efficient enrichment of the target gas, thus significantly shortening the response time. In addition, the silicon micropillar array has a larger specific surface area; the gas-sensitive material can be deposited not only on the bottom and top surfaces but also on the side surfaces of the square micropillars, which remarkably increases the number of active sites and further enhances the NH_3_ adsorption capacity. Furthermore, the spatial supporting effect of the silicon micropillar array can effectively suppress the agglomeration of the material, ensure the integrity of the pore structure, and improve the efficiency of electron transport as well as the stability of detection [41,42].

## 4. Conclusions

In summary, in this work, MOF-808 and MOF-818 were synthesized, and then mixed with reduced graphene oxide (rGO) to fabricate MOF/rGO composites as gas-sensitive materials for NH_3_ detection. First, interdigital electrodes were employed as the gas sensor substrates. By regulating the electrode width and the types of gas-sensitive materials, the optimal electrode width and gas-sensitive material were successfully screened out. The experimental results demonstrated that when the gas-sensitive materials coated on the sensor surface were identical, the gas sensor with an electrode width of 253.1 μm exhibited the shortest response time to 40 ppm NH_3_. Among the three gas-sensitive materials (rGO, MOF-808/rGO, and MOF-818/rGO), the sensor modified with MOF-808/rGO delivered the best sensing performance. This was attributed to the larger Brunauer–Emmett–Teller (BET) specific surface area and more gas adsorption active sites of MOF-808. The MOF-808/rGO sensor with an electrode width of 253.1 μm presented a response time of 197 s and a Response(%) of 6.91% to 40 ppm NH_3_. Subsequently, plasma treatment was applied to this sensor to introduce more surface defects and oxygen-containing functional groups. A comparison of the sensing performance before and after plasma treatment indicated that the treated sensor achieved a shorter response time of 168 s and a higher Response(%) of 9.67%. Then, the silicon micropillar array was introduced as the sensor substrate, with MOF-808/rGO serving as the gas-sensitive material. By adjusting the silicon micropillar array arrangement period, an NH_3_ sensor with excellent sensing performance was successfully prepared. The experimental results showed that when the micropillar side length was 5 μm and the inner micropillar was 8.2 μm, the sensor exhibited a response time of 159 s and a Response(%) of 18.3% to 40 ppm NH_3_. In comparison with the optimal interdigital electrode sensor without plasma treatment, the response time was shortened by 19.3%, and the Response(%) was increased by 1.65 times. This indicated that the introduction of the silicon micropillar array could enhance the sensing performance, which benefited from the abundant gas flow channels and larger specific surface area of the micropillar structure. Finally, the sensor with the best performance was also subjected to surface modification via oxygen plasma bombardment. It was found that after plasma treatment, the silicon micropillar array sensor achieved an optimal response time of 75 s in the detection of 40 ppm NH_3_. Overall, this work verified that the MOF-808/rGO sensor based on the silicon micropillar array substrate exhibited excellent detection performance for NH_3_. This was ascribed to the synergistic effect between the adsorption capacity of MOF-808 and the electrical conductivity of rGO, as well as the unique 3D structure of the silicon micropillar array. These results provide a feasible strategy for room-temperature NH_3_ detection.

To comprehensively evaluate the performance of the developed MOF-808/rGO silicon micropillar array sensor, a comparative analysis of key metrics, including response time and limit of detection (LOD), was conducted against representative room-temperature NH_3_ gas sensors reported in the recent literature (Table 3). The results demonstrate that the proposed 3D silicon micropillar array architecture offers an exceptional balance between response kinetics and sensitivity. While certain materials, such as CNTs@MoS_2_, achieve rapid response times, they often suffer from significantly higher LODs in the double-digit ppm range. Conversely, while UIO-66 is a recognized framework, it exhibits sluggish response times compared to our device. By integrating the MOF-808/rGO composite with a 3D micropillar substrate, our sensor achieves a competitive response time of 75 s to 40 ppm NH_3_ and an impressive LOD of 0.312 ppm. This performance surpasses that of conventional rGO-PANI and UIO-66 sensors, confirming that our structural engineering approach provides a superior strategy for achieving highly sensitive and rapid NH_3_ detection at room temperature.

## Figures and Tables

**Table 1:** Specific Surface Area and Pore Data of MOF Materials.

Samples	BET Surface Area (m^2^/g)	Total Pore Volume (cm^3^/g)	Average Pore Width (nm)
MOF-808	851.32	0.64	3.05
MOF-818	613.17	0.41	1.84

**Table 2:** Comparison of initial resistance and Response(%) of silicon micropillar array MOF-808/rGO sensors before and after plasma treatment.

Samples	R_0_/kΩ (30 s Plasma Treatment)	R_0_/kΩ (120 s Plasma Treatment)	Response(%) (30 s Plasma Treatment)	Response(%) (120 s Plasma Treatment)
Period-1	17.47	21.36	10.79%	10.12%
Period-2	15.61	18.89	6.76%	7.08%
Period-3	19.45	27.91	6.17%	7.22%
Period-4	22.81	27.74	12.40%	17.02%
Period-5	29.62	31.08	9.92%	6.58%

**Table 3:** Comparison of room-temperature NH_3_ sensing performance with recently reported sensors.

Sensor Material	Substrate/Structure	NH_3_ Concentration	Response Time	Limit of Detection (LOD)
MOF-808/rGO (This work)	3D Silicon Micropillar Array	40 ppm	75 s	0.312 ppm
rGO-PANI [43]	Interdigitated electrodes (IDE)	100 ppm	97 s	5 ppm
Cu_3_(HHTP)_2_ [44]	NA	100 ppm	82 s	0.5 ppm
CNTs@MoS_2_ [45]	Interdigitated silver electrodes	500 ppm	36 s	10.33 ppm
UIO-66 [46]	Interdigitated electrodes (IDE)	50 ppm	136 s	NA

## Data Availability

The raw data of this study are included in the paper. For further inquiries, please contact the corresponding author.

## Supplementary Materials

The following supporting information can be downloaded at: https://www.mdpi.com/article/10.3390/s26103216/s1, Figure S1: The gas-sensing measurement system; Figure S2: EDS images of (a) MOF-818; (b) MOF-808; Figure S3: XRD patterns of MOF-808 and MOF-808/rGO; Figure S4: Pore size distributions of MOF-808 and MOF-818; Figure S5: (a_1_–a_3_) Response–recovery curves of the interdigitated electrode-based rGO sensors toward 40 ppm NH_3_; (b_1_–b_3_) Response–recovery curves of the interdigitated electrode-based MOF-818/rGO sensors toward 40 ppm NH_3_; (c_1_–c_3_) Response–recovery curves of the interdigitated electrode-based MOF-808/rGO sensors toward 40 ppm NH_3_; Figure S6: (a_1_–b_1_) Repeatability and (a_2_–b_2_) concentration gradient curves of interdigitated electrode-based MOFs/rGO sensors toward 40 ppm NH_3_; Figure S7: Respons–recovery curves of the interdigitated electrode-based MOF-808/rGO sensors to 40 ppm NH3 following oxygen plasma treatment for (a1–c1) 30 s and (a2–c2) 120 s; Figure S8: Concentration gradient curves of the silicon micropillar array MOF-808/rGO sensors toward 40 ppm NH_3_; Figure S9: Response–recovery curves of plasma-treated silicon micropillar array MOF-808/rGO sensors toward 40 ppm NH_3_; Figure S10: Schematic illustration of the energy-band structure and Schottky barrier modulation at the Sn/rGO interface before and after NH_3_ exposure; Figure S11: High-resolution XPS O 1s spectra of the MOF-808/rGO composite before (a) and after (b) oxygen plasma treatment; Table S1: Deconvolution parameters (relative percentages and absolute areas) of the O 1s spectra for the MOF-808/rGO composite before and after oxygen plasma treatment.
