# Supplementary material for: Fabrication and Enhancement of the Gas Sensing Characteristics of Silicon Micropillar NH3 Sensors Based on MOF-808/rGO Nanocomposites at Room Temperature
Source: Sensors (Basel). 2026 May 19;26(10):3216. doi: 10.3390/s26103216 (PMC13210459; doi:10.3390/s26103216)
Supplement: Supplementary file 1 [file sensors-26-03216-s001.zip › sensors-4284768-supplementary.pdf]

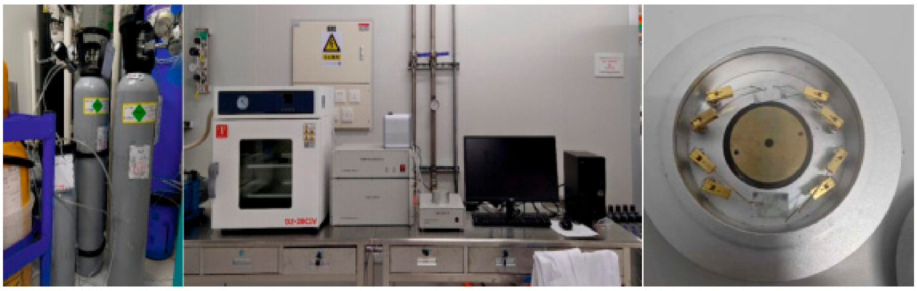

FigureS1. The gas-sensing measurement system.

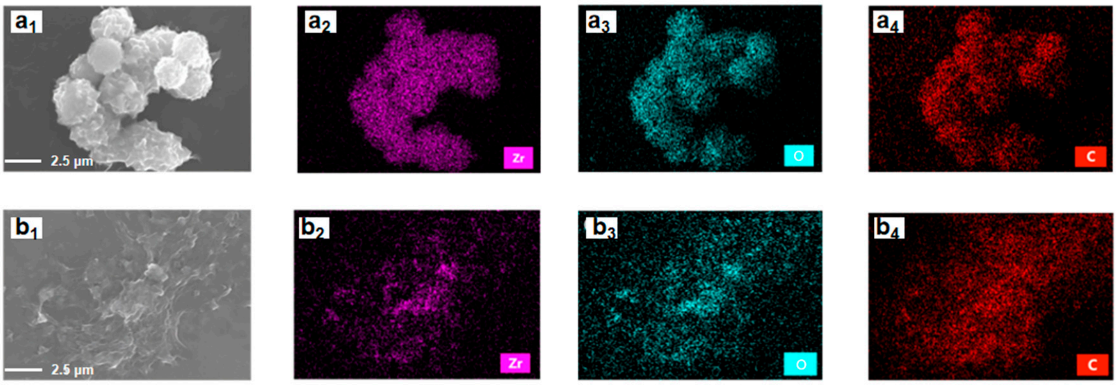

FigureS2. EDS images of (a)MOF-818;(b) MOF-808.

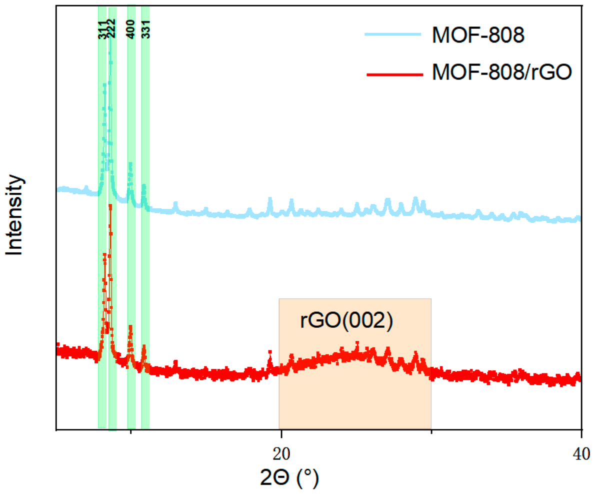

Figure S3. XRD patterns of MOF-808 and MOF-808/rGO.

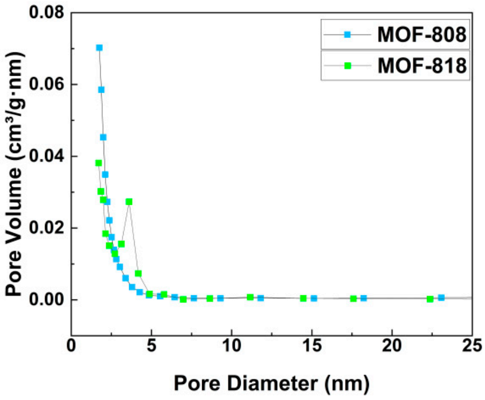

Figure S4. Pore size distributions of MOF-808 and MOF-818.

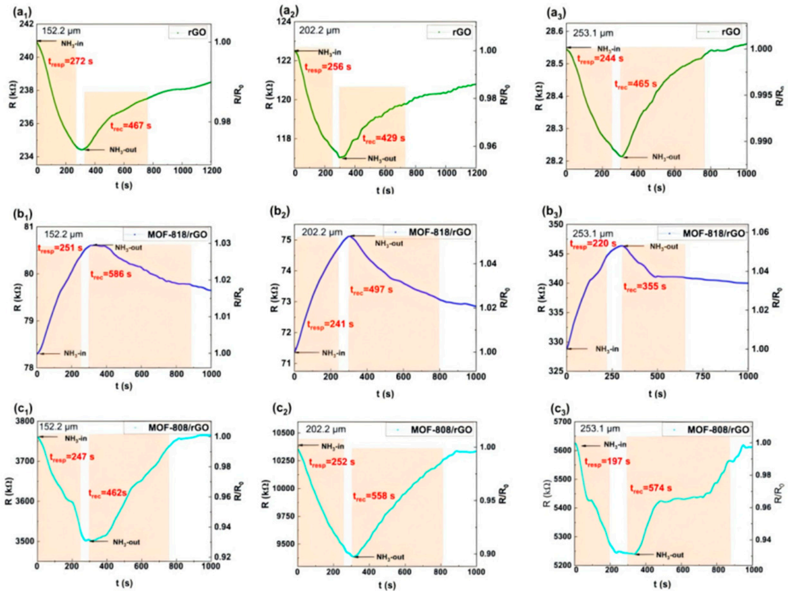

Figure S5. (a1–a3) Response–recovery curves of the interdigitated electrode-based rGO sensors toward 40 ppm NH_3;(b1–b3) Response–recovery curves of the interdigitated electrode-based MOF-818/rGO sensors toward 40 ppm NH_3; (c1–c3) Response–recovery curves of the interdigitated electrode-based MOF-808/rGO sensors toward 40 ppm NH_3.

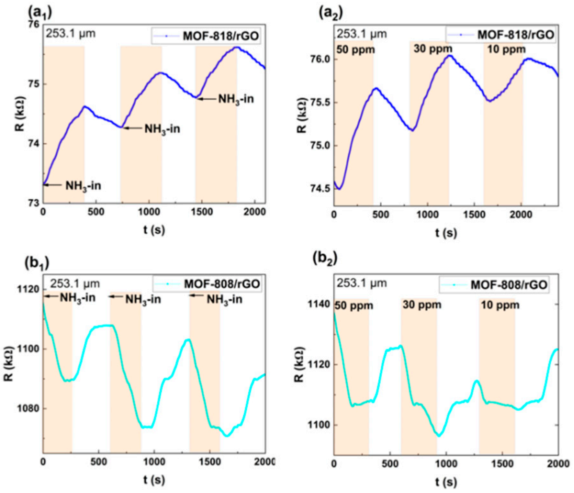

Figure S6. (a1-b1)Repeatability and (a2-b2)concentration gradient curves of interdigitated electrode-based MOFs/rGO sensors toward 40 ppm NH_3.

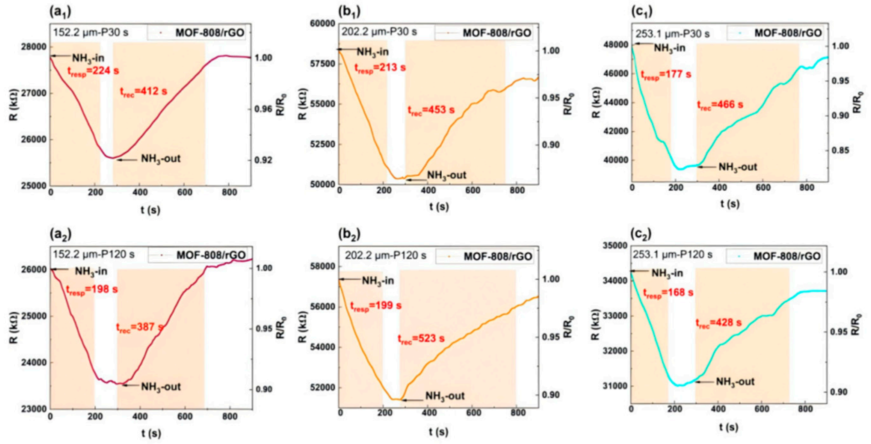

Figure S7. Respons–recovery curves of the interdigitated electrode-based MOF-808/rGO sensors to 40 ppm NH_3 following oxygen plasma treatment for (a1–c1) 30 s and (a2–c2) 120 s.

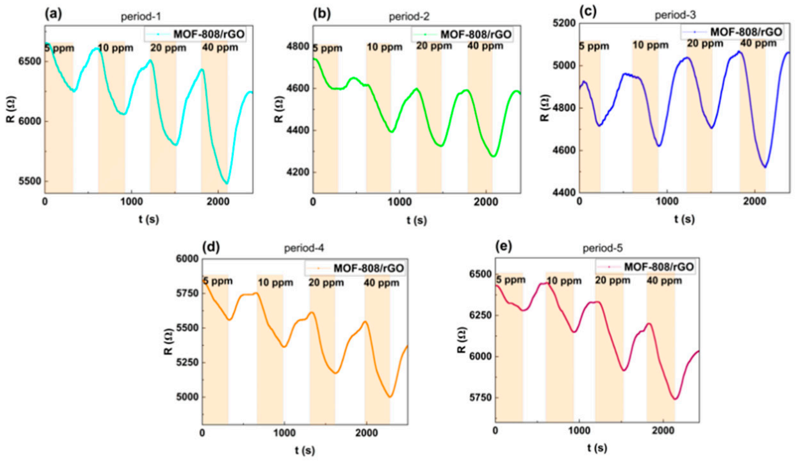

Figure S8. Concentration gradient curves of the silicon micropillar array MOF-808/rGO sensors toward 40 ppm NH_3.

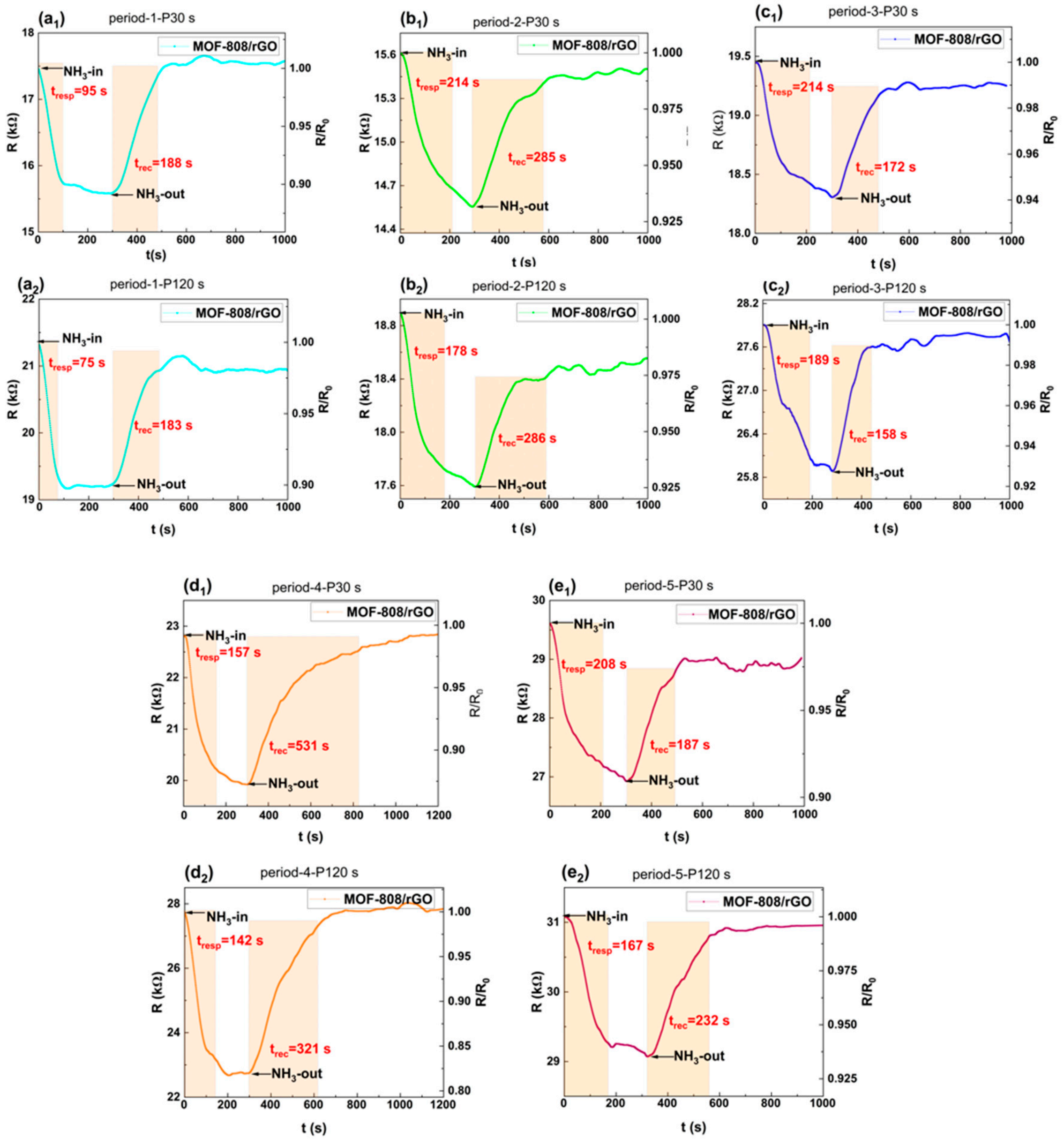

Figure S9. Response–recovery curves of plasma-treated silicon micropillar array MOF-808/rGO sensors toward 40 ppm NH_3.

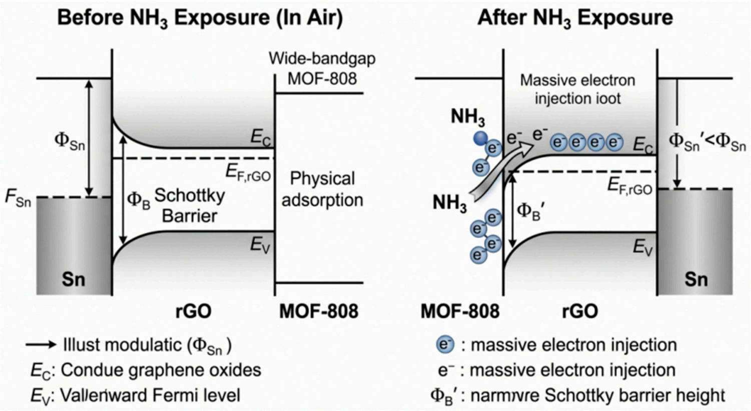

Figure S10. Schematic illustration of the energy-band structure and Schottky barrier modulation at the Sn/rGO interface before and after NH_3 exposure.

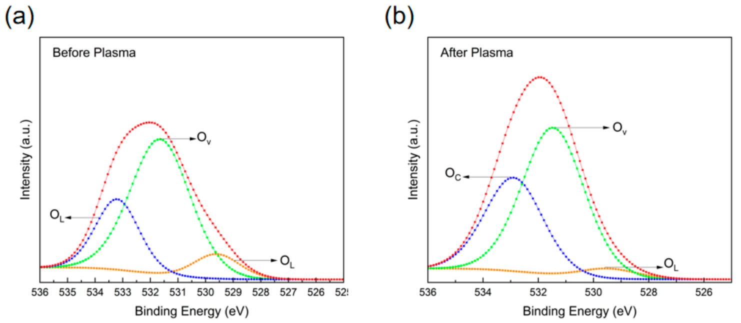

Figure S11. High-resolution XPS O 1s spectra of the MOF-808/rGO composite before (a) and after (b) oxygen plasma treatment.

Table S1. Deconvolution parameters (relative percentages and absolute areas) of the O 1s spectra for the MOF-808/rGO composite before and after oxygen plasma treatment.

	Relative percentage(before plasma)	Relative percentage(after plasma)	Area (before plasma)	Area (after plasma)
O_L	9.22%	3.22%	297.9	164.6
O_V	65.89%	60.25%	2131.8	2498.7
O_C	24.89%	35.83%	806.1	1487.3
